# Adaptation to Freshwater in Allis Shad Involved a Combination of Genomic and Epigenomic Changes

**DOI:** 10.1007/s00239-025-10253-9

**Published:** 2025-06-02

**Authors:** Paulo Pereira, Sandra Afonso, António Múrias, Miguel Carneiro, Stephen J. Sabatino

**Affiliations:** 1 CIBIO - Centro de Investigação em Biodiversidade e Recursos Genéticos, Universidade do Porto, Vairão, Portugal; 2 BIOPOLIS - Program in Genomics, Biodiversity and Land Planning, CIBIO, Vairão, Portugal; 3 Departamento de Biologia, Faculdade de Ciências, Universidade do Porto, Porto, Portugal

**Keywords:** Adaptation, DNA methylation, Epigenetics, Freshwater

## Abstract

Epigenetic modifications are one of the evolutionary mechanisms that allow individuals and populations to adapt to environmental changes. However, the relative importance of epigenetic versus genetic changes in adaptation and how they may interact remains poorly understood. Here, we investigate the role of DNA methylation in adaptation by studying a population of Allis shad (*Alosa alosa*) that evolved a completely freshwater life history approximately 70 years ago and the anadromous one that founded it. Using reduced representation bisulfite sequencing, we identified 227 differentially methylated regions (DMRs) between them, overlapping known important genes for freshwater adaptation, such as ATP2B4, PRLH2, and KCNF1A. Enrichment analysis of GO terms suggested that genes in the identified DMRs play key roles in neural, growth, and developmental functions, which is concordant with previous studies on adaptation to freshwater in this species and genus. Using pool-seq data from an earlier study, we then tested if the DMRs for freshwater shad found here overlapped genomic outlier regions that may be under genetic selection in three independently evolved, freshwater populations (including the one studied here). Our analysis showed that the DMRs identified here fall broadly outside genomic regions under natural selection. However, 45% of these were associated with CpG > TpG deamination events in DMRs, a mutation tightly linked with DNA methylation. Our study illustrates that both genetic and epigenetic mechanisms are important during the initial stages of adaptation in this system. It also supports the hypothesis that methylation may generate polymorphism that fuels adaptive evolution.

**Supplementary Information:**

The online version contains supplementary material available at 10.1007/s00239-025-10253-9.

## Introduction

The mode and tempo at which species adapt to new conditions have been the subject of extensive research for many years (Simpson 1984). Contrary to early expectations (Darwin 1859), recent studies have shown that adaptation can occur over just one or a few generations (Messer & Petrov 2013; Terekhanova et al. 2014). Natural selection can act on new, or existing (i.e., ancestral) mutations that impact fitness traits, resulting in adapted phenotypes (Cayuela et al. 2020; Perfeito et al. 2007). While ancestral polymorphism can fuel adaptation (Colosimo et al. 2005; van’t Hof et al. 2019), in some cases, there appears to be a weak or nonexistent relationship between genetic variation and adapted phenotypes (Charmantier et al. 2008; Riis et al. 2010; Trussell & Smith 2000). In such cases, species’ responses to natural selection may be tied to phenotypic shifts that do not rely on genetic change, such as phenotypic plasticity and epigenetic change (Larsen et al. 2011).

There is growing evidence that epigenetic control of gene expression can play a central role in adaptation (Bollati & Baccarelli 2010; Jaenisch & Bird 2003; Venney et al. 2021, 2022). Epigenetics broadly includes a set of heritable (either meiotically or mitotically) mechanisms that influence gene regulation without requiring changes in the underlying DNA sequence (Bird 2007; Jaenisch & Bird 2003). These include histone modifications through acetylation, phosphorylation, and methylation, altering the chromatin structure (Bannister & Kouzarides 2011; Rice & Allis 2001), and non-coding RNAs (e.g., microRNAs) that mediate gene expression (Jackson & Standart 2007). Epigenetics also includes DNA methylation, which is a modification of the DNA base composition where a methyl group is added to the cytosine, forming 5-methylcytosine (5mC). Although vertebrate genomes are generally highly methylated, mainly in the context of CpG dinucleotides, the distribution of methylation markedly varies across the genome, and CpG methylation is rare in promoter regions (Al Adhami et al. 2022). Importantly, DNA methylation in promoters and other regions (e.g., enhancers, first introns and exons), can control gene expression and thus can alter phenotypic plasticity and shape fitness-related traits (Anastasiadi et al. 2018; Basu et al. 2022; Brenet et al. 2011; Cho et al. 2022; Greenberg & Bourc’his, 2019; Yin et al. 2017). Another intriguing way methylation may impact adaptive evolution is because 5mC tends to rapidly deaminate to thymine (Cooper et al. 2010; Cooper & Krawczak 1989; Sved & Bird 1990). This accelerated mutational event can result in the creation of genetic diversity, which can then be subsequently targeted by natural selection (Cooper et al. 2010). While the potential for epigenetic changes to play key roles in adaptation is clear, the extent to which they do, and whether they act independently or in concert with genetic changes, remains a largely open question.

DNA methylation is involved in adaptation to different ecological conditions in a variety of species, including variation in temperature and salinity in marine species (Blondeau-Bidet et al. 2023; Cayuela et al. 2020; Liew et al. 2020; Trautner et al. 2017; Varriale & Bernardi 2006; Venney et al. 2021; Vogt 2018). These findings include populations of three-spined stickleback (Artemov et al. 2017; Heckwolf et al. 2020; Hu et al. 2021; Hu & Barrett 2023; Metzger & Schulte 2018; Ord et al. 2023; Smith et al. 2015) that have evolved completely freshwater life histories after adapting to lake habitats. In stickleback, they found that differences in DNA methylation was associated with genes involved with traits that are involved in adapting to a freshwater life history, such as osmoregulation and development, and that are likely under strong genetic selection (Artemov et al. 2017; Heckwolf et al. 2020; Hu et al. 2021; Hu & Barrett 2023; Metzger & Schulte 2018; Ord et al. 2023; Smith et al. 2015). However, little is known about how general these findings are across other anadromous species with freshwater-adapted populations concerning the genes involved, and the relationship between DMRs and allele frequencies differences for adaptive traits.

The Allis shad (*Alosa alosa,* Linck 1790) presents an extraordinary opportunity to explore the role of epigenetics in rapid adaptation. The Allis shad is an anadromous species distributed across the northeastern Atlantic. Over the past century, dam construction in three Portuguese rivers where they spawn resulted in the genesis of new landlocked populations. Individuals of these populations cannot reach the sea and are no longer anadromous. This semi-natural experiment provides an excellent laboratory for studying how species can evolve new life history traits in just a few generations. The adaptation of these populations to a completely freshwater life cycle entails strong shifts in their biology. Initially, this transition involved physiological changes necessary to maintain osmotic homeostasis in freshwater throughout its life cycle. Among other differences, landlocked individuals also no longer need the swimming capacity or fat reserves to make long-distance migrations like their anadromous ancestors (Aprahamian et al. 2003; Velotta et al. 2018) and have undergone a dietary shift (Aprahamian et al. 2003; Collares-Pereira et al. 1999). This drastic shift in life history and ecology has resulted, at least in part, in freshwater shad exhibiting a sharp decrease in size and number of gill rakers (Aprahamian et al. 2003). Previous work on the system has demonstrated key molecular changes that are important to the establishment of these freshwater populations. For instance, when testing for genomic regions that show convergent evolution to freshwater adaptation, Sabatino et al. (2022) found that nonsynonymous mutations within ATPase-α1.1b show convergent evolution between two of the four lineages that have independently adapted to a complete freshwater life history. However, levels of convergent evolution among landlocked populations in Portugal are modest, hinting toward the existence of multiple paths leading to adaptation (Sabatino et al. 2022).

**Fig. 1:**
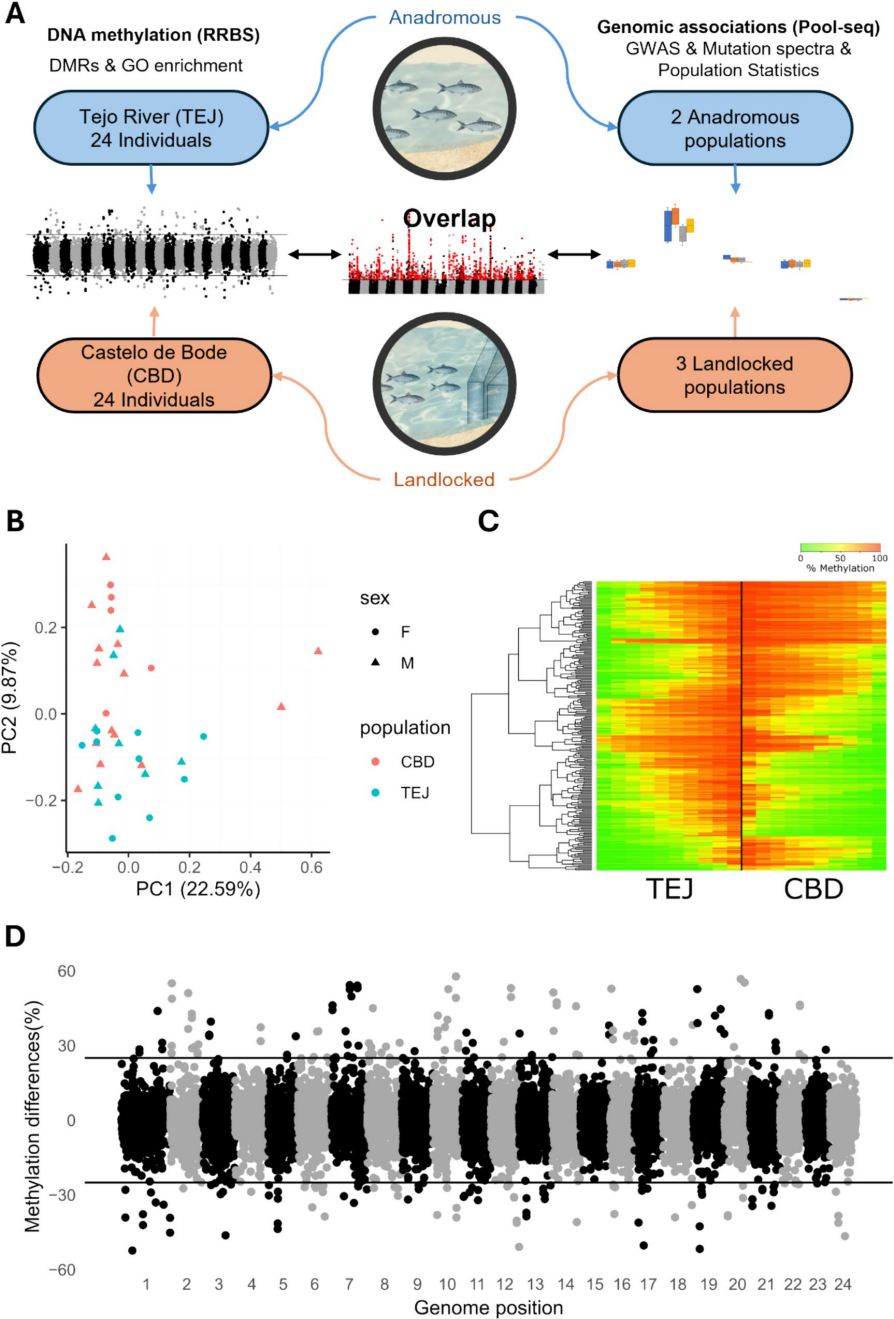
**A** Schematic representation of the approach followed within this study. We used methylation data (this study) together with Pool-seq information (Sabatino et al. 2022) to explore the role of the epigenome and genome in the adaptation to freshwater. Finally, we use the results from both components to investigate overlaps between data sources. **B** First two axes of the principal component analysis performed on the methylation variation present on the 20,680 windows retained in our analysis between ecotypes. Points in light blue represent the Tejo (anadromous) population, while red points represent Castelo de Bode (landlocked). **C** Heatmap representation of the 227 differentially methylated regions (rows) identified in our study. To reduce graphical noise from missing data, we sampled 10 individuals for each gene/population combination minimizing overall variance and maximizing fidelity. Due to this transformation, columns on the heatmap lose correspondence with individuals, and as such, were sorted to highlight differences between clusters – see supplementary Fig. 1 for the untransformed figure. **D** Manhattan plot of methylation differences across the genome. Each dot represents one of the 20,680 windows analyzed. Positive values correspond to hypermethylated regions while negative values correspond to hypomethylated regions in Tejo compared to Castelo de Bode. Horizontal lines represent the 25% difference cut-off

Here, we investigate if epigenetics, and more precisely, DNA methylation have played a role in the adaptation of anadromous *A. alosa* to a freshwater life cycle. To do so, we conducted reduced representation bisulfite sequencing (RRBS) on samples obtained between 1998 and 1999 from both the landlocked population in the Portuguese reservoir of Castelo de Bode dam (CBD; built in 1951) and its anadromous founder in the Tejo River (TEJ). Using this recent (approximately 11 generations), rapid adaptive event as a model, we aim to test if and how DNA methylation affects adaptation. More specifically (see Fig. 1A for a schematic overview), we aim to: 1) identify regions of the genome that show different levels of methylation (differentially methylated regions or DMRs) between the two life histories; 2) evaluate if DMRs are associated with genes known to be involved in freshwater adaption in marine species in general, and contrast with regions under genetic selection in this species, and lastly; 3) given that methylated Cytosines in CpG context are often deaminated, leading to Cytosine to Thymine transitions at an accelerated rate, we aim to evaluate if the ancestral genetic diversity created through this process is a target of natural selection during adaptation.

## Materials and Methods

### Identifying Differentially Methylated Regions Associated with the Transition to Freshwater

#### Sample Collection and Library Preparation

To compare methylation patterns between ecotypes of shad, we used samples of 48 individuals from one freshwater (24 collected in 1999; 6 females; 18 males) and one anadromous population (12 collected in 1998; 12 collected in 1999; 14 females; 9 males; 1unknown) of *A. alosa* that inhabit the Tejo hydrographic basin in Portugal. The anadromous ecotype of Rio Tejo (TEJ) sampled spawns below the Castelo de Bode dam and spends up to seven years at sea feeding, while those of the freshwater ecotype complete their entire life cycle in the Castelo de Bode reservoir (CBD). Samples of each population were collected at the start of the breeding season of each ecotype and stored at -80ºC. Anadromous individuals were caught while they were migrating upstream, presumably to spawn. Due to tissue-specific differences in methylation (Lokk et al. 2014) and due to the availability of sampled tissues, we used muscle to measure differences in methylation between populations. While it is hard to predict which tissue would be most appropriate to target in the present scenario, muscle is involved in a wide variety of life and fitness-related traits in shad. Genomic DNA was extracted using EasySpin commercial kit (Citomed), following the manufacturer’s protocol, with an extended lysis period (overnight) to maximize DNA yield. DNA quality and quantity were accessed through Nanodrop and Qubit (High Sensitive DNA kit).

For library preparation, we followed a modified version of the multiplex reduced representation bisulfite sequencing mRRBS (Boyle et al. 2012). Briefly, per sample, 200 ng of DNA was digested with restriction enzyme MspI (recognition site $$\begin{array}{*{20}c} {C \vee CGG} \\ {GGC \wedge C} \\ \end{array}$$; New England Biolabs) at 37 ºC for 16 h without inactivation. Gap filling and A-tailing were simultaneously achieved using Klenow Fragment (exo -; Thermofisher) with a dATP-rich dNTP mixture (10 mM dATP, 1 mM dBTP; ThermoFisher), incubated at 30 ºC for 20 min followed by 37 ºC for 20 min. Unincorporated dNTPs were removed using a 2 × bead cleanup method (Ampure XP; Beckman Coulter) and gap-filled, end-prepped fragments were eluted in 20 μl of elution buffer (New England Biolabs). Truseq adapters (Truseq DNA Single Index set A & B; Illumina) were incorporated using T4 ligase (ThermoFisher) by incubating overnight at 16ºC and subsequently cleaned using Ampure XP beads (Beckman Coulter) with a 2:1 bead-to-sample volume ratio. Bisulfite Conversion was carried out using the EpiTect Bisulfite Kit (Qiagen) following the manufacturer’s protocol for FFPE-preserved samples. To maximize efficiency, two consecutive rounds of conversion were conducted. Finally, converted fragments were PCR amplified using EpiMark® Hot Start Taq DNA Polymerase (New England Biolabs) with Truseq Primers, followed by a bead size selection step with a 1.2:1 bead-to-library volume ratio. To increase library molarity, four extra PCR cycles were performed using KAPA Library Amplification Kit (Roche). In addition, two extra bead cleanup steps were conducted (1:1 bead to library volume ratio) to remove any trace of adapter dimer. Library quality control was performed by qPCR (KAPA Library Quantification Kits—Complete kit, BioRad iCycler only; Roche) and TapeStation HS D5000 (Agilent). An in silico digestion with MspI was performed to establish the reduced representation genome size. Libraries were sequenced in a Novaseq 6000 with 150 paired-end reads.

#### RRBS Quality Assessment and Mapping

The quality of the resulting DNA sequence reads was assessed with FASTQC (v0.11.9 Babraham Bioinformatics). Specifically, we evaluated the depletion of cytosines together with the overall quality of the libraries. A noticeable overrepresentation of poly-G in read2 indicated a relatively poor run performance in the reverse strand. We, therefore, performed paired and single-read mapping steps (see below). Reads were adapter and quality trimmed using TrimGalore (v0.6.5 Babraham Bioinformatics), using a minimum quality of 20 to trim read ends and trimming all bases that overlap with adapter sequence. Finally, only reads longer than 36 bp were retained. The RRBS option was used to remove gap-filled bases from the analysis. At this point, samples with an estimated coverage lower than 10X were removed from the analysis.

Filtered-trimmed reads were then mapped to the *Alosa alosa* genome (AALO_Geno_1.1, GenBank accession number GCA_017589495.2) using Bismark 0.22.3 (Krueger & Andrews 2011) using a two-step approach (Supplementary Table 1). First, an in silico bisulfite treatment of the genome was performed. Due to the loss of strand complementarity through cytosine deamination and subsequent conversion to thymine, two different genome strands were constructed and treated using *bismark_genome_preparation*. Secondly, reads were mapped to both strands using Bismark, allowing soft clipping through the *–local* option. Since we detected some issues in read2, we allowed Bismark to do a non-directional mapping where mapping against the original 5’-3’ and 3’-5’ bisulfite-treated strands and their reverse complements was permitted. Furthermore, we performed a second round of mapping using all unmapped reads and treating them as a single-ended libraries.

Cytosine methylation status was independently estimated for both paired and single-end datasets using *bismark_methylation_extractor* for all configurations (CpG, CpH, and CHH) with the *comprehensive* flag. However, we only consider methylation at CpG sites here. Finally, for each sample, per CpG methylation percentage and coverage were estimated using *bismark2bedGraph* providing the extracted methylation calls for both paired and single-end datasets, effectively combining both datasets.

#### Identification of Differentially Methylated Regions (DMRs)

We then identified differentially methylated regions that can be associated with the transition from an anadromous to freshwater life history. To estimate differences in methylation between the anadromous and landlocked populations, we used methylKit (Akalin et al. 2012). Sites covered by either fewer than three reads or more than the top 1% of coverage (per individual) were discarded as they either had low support or were likely the result of PCR bias. Coverage was then normalized across samples, and methylation was summarized into 2000 bp windows with a step size of 1000 bp. To be conservative, we only considered windows in the analysis where at least ten valid CpG sites had to be present in the window, and the window had to be present in at least half (n = 10) of the retained samples of each group (anadromous and landlocked). We limited the analysis to windows that overlapped the main 24 chromosomes of *A. alosa*, which represent 97.79% of the genome. This decision was motivated by two observations. First, the data mapped to unplaced scaffolds tended to differ from the main chromosomes (e.g., abnormally high variance), which might skew the analysis. Second, genes are rare in unplaced scaffolds (including only 142 out of 23,478 protein-coding genes) and should not severely impact our analysis. To evaluate the discriminatory power of our data to distinguish differences between the two life histories, we performed a scaled PCA using the *PCASamples* function (methylkit). Differentially methylated regions between anadromous and landlocked individuals were then estimated using the function *calculateDiffMeth* (methylkit) using the logistic regression-based test (Akalin et al. 2012). We added the individual sexes as covariates to the logistic regression to control for potential differences that might have arisen due to sex-related methylation changes. Differentially methylated regions were considered those with at least a q-value of 0.05 and a difference in methylation percentage of at least 25% between the two groups.

#### Gene Ontology Enrichment Analysis

We investigated whether genes overlapping differentially methylated regions are enriched for specific functions. To do so, we first limited the available proteome in GenBank to the largest isoform available for each gene and annotated it with the *annotate your proteome* function from STRINGS (Jensen et al. 2009; https://string-db.org/). GoSEQ (Young et al. 2010) was then used to detect enriched GO (Gene ontology) terms using the Wallenius method to control for potential bias between enrichment and gene length.

### Measuring the Association Between DMRs and Regions of the Genome Under Genetic Selection

#### Scans for Genomic Outlier Regions Under Genetic Selection

After identifying differentially methylated regions between the two life histories, we set up to determine if these DMRs are associated with regions under genetic selection. We hypothesized that if there is an overlap between DMRs and genetic outlier regions, then the differences in methylation are likely due to the selective pressure on the genomic sequence rather than an adaptive mechanism. To do so, we measured the overlap between differentially methylated regions and regions potentially under genetic selection. We utilized previously published (NCBI BioProject: PRJNA649106; Sabatino et al. 2022) whole-genome sequencing data from five populations (two anadromous and three landlocked) and re-mapped it to the *Alosa alosa* genome used in this study (AALO_Geno_1.1, GenBank accession number GCA_017589495.2) using BWA-MEM (Li 2013) followed by a local realignment using *GATK RealignerTargetCreator* and *IndelRealigner* (McKenna et al. 2010). FreeBayes v0.9.21 (Garrison & Marth 2012) was used to independently call variant positions with the following settings: use-best-n-alleles 4, pooled-discrete, min-coverage 10, min-repeat-entropy 1, min-mapping-quality 60, min-base-quality 20, use-mapping-quality, max-coverage 100. The FreeBayes dataset was filtered to retain only biallelic SNPs with a quality score greater than 20. Filtered mpileups were then reconstructed from the final set of SNP loci using a custom Python script. These pileups were used to estimate minor allele frequencies using *snape-pooled* (Raineri et al. 2012) folded SFS using the informative prior theta of 0.01 and a prior genetic difference between reference and population of 0.1. Finally, minor allele frequencies were averaged across windows of 20 SNPs, with a step size of 10 SNPs, using a Python script and used in PCAdapt (Luu et al. 2017) to detect genomic regions associated with local adaptation, with the following parameters: number of clusters (k) of 4 and minimum minor allele frequency (min.maf) set to 0. In *A. alosa*, population structure reflects life history changes (Sabatino et al. 2021, 2022). Therefore, PCAdapt becomes a suitable tool for exploring genomic regions involved in this transition.

#### Determining Overlaps Between DMRs and Genomic Outlier Regions

To evaluate the possible interplay between differentially methylated regions and candidate regions under selection, we looked at the overlap between the top 1% of outlier windows from PCAdapt and the 227 identified differentially methylated regions (Supplementary Table 2). Due to the different sizes in DMRs and candidate regions, we counted both partial and complete overlaps.

### Estimating the Contribution of Deamination Events to Standing Levels of Polymorphism and Adaptive Potential

#### Measuring the Mutational Spectra

To understand if the nucleotide diversity generated through 5mC deamination to Uracil-Thymine had a significant role in the transition from anadromous to freshwater, we looked at the mutation spectrum between the two populations. To do so, we used a custom Python script that used the minor allele frequencies from the pool-seq data and compared transitions between all pairs of populations. Specifically, in each comparison, all SNPs with a delta allele frequency of 0.5 were allocated to the distinct types of transition and transversion mutations (A > C; A > G; A > T; C > A; C > G; C > T; CpG > TpG; TpG > CpG). To summarize and visualize the results, strand complements were considered as the same mutation (e.g., T > G was considered the same as A > C, and the same is true for the dinucleotide motifs; for example, CpG > CpA was collapsed in the CpG > TpG category). We then focused our analysis on mutations between four distinct groups: freshwater to freshwater, freshwater to anadromous, anadromous to freshwater, anadromous to anadromous. Statistical differences between these directions were assessed with an ANOVA followed by Tukey’s Honest Significant Difference post hoc test.

#### Investigating the Population Genetics of DMRs Versus Other Regions of the Genome

We evaluated how the differences in methylation and 5mC deamination correlate with potential population genetic diversity to test whether the patterns identified were driven by increased nucleotide diversity (π) or selection. To do this, we estimated Tajima’s Pi to assess how this measure of nucleotide diversity correlates with these two results using *Popoolation* (Kofler et al. 2011) *Variance-Sliding.pl* script with the following options: window size: 20 kb; step size: 5 kb; min-count: 2; min-qual: 20; pool-size: twice the number of individuals in the pool; min-coverage: 5; max-coverage: twice the genome-wide average; min-covered-fraction: 0.2. Average π per life history was computed and to evaluate regions of the genome with an elevated accumulation of genetic diversity between life histories, we computed Δπ as the difference in the average between anadromous and landlocked.

Recently, Ord et al., (2023) argued that sites with relaxed methylation maintenance are also under weaker selective constraints, leading to increased nucleotide diversity (Ord et al. 2023). Given that the CBD freshwater population is just tens of generations old, it is unlikely that most of the observed CpG-TpG transitions resulted from relaxed selection at mCpG sites, as was observed in the much older freshwater stickleback populations (Ord et al. 2023). Alternatively, these transitions were ancestral events that occurred in the anadromous populations that then colonized CBD. If the alternative is true, and these SNPs are ancestral, we do not expect a significant increase in nucleotide diversity in DMRs since there has not yet been enough time to generate nucleotide diversity in the freshwater population. To test this hypothesis, we used a modified version of the approach outlined in Ord et al. 2023. Briefly, CGmap-tools v0.1.2 (Guo et al. 2018) was used to identify C-T/G-A SNPs in our Bismark maps. Positions that appeared in at least one individual and had five or more reads covering them were removed from the Bismark coverage files across all individuals. We then used methylKit (Akalin et al. 2012) on the filtered dataset to identify differentially methylated cytosines between life history groups. To help ensure our results were comparable to the original study, we incorporated different thresholds, testing for the number of individuals (between 3 or 5 individuals covering the same position) and coverage at position (minimum 3 or 5 × coverage). Finally, we utilized variance-at-position.pl from the Popoolation toolkit (Kofler et al. 2011) to estimate several statistics (π, Watterson’s *Θ*, and Tajima’s D) for different types of positions (hypermethylated in freshwater, hypomethylated in freshwater or non-differentially methylated). Population statistics were calculated for each chromosome, where each type of position was categorized as a “gene” so that a single measure of each category was obtained per chromosome. To avoid bias due to differences in the number of positions between the differentially methylated and non-differentially methylated categories, a comparable subset of the latter was used. We run variance-at-position.pl the following parameters: min-count: 2; min-qual: 20; pool-size: twice the number of individuals in the pool; min-coverage: 5; max-coverage: twice the genome-wide average.

#### Investigating the Role of mCpG Deamination in Adaptation

Given the high rate of deamination of 5-methylcytosines into thymine, which is the DNA motif with the highest mutation rate in vertebrate genomes (Cooper & Krawczak 1989), we postulate that past differences in DNA methylation and subsequent historical deamination events could have fostered standing genetic diversity. This diversity can then be recruited by selection as an adaptive response to environmental change. As a proxy to a very complex process, we measure how often regions of the genome previously identified to be under selection contain CpG-TpG deamination events with a high (0.5) difference in allele frequency between landlocked and anadromous populations. In contrast, these strong shifts in allele frequency in CpG-TpG transitions could also be responsible for an overestimation of DMRs. To test for their effect, we further overlap our differentially methylated regions (DMRs) with the same set of CpG-TpG transitions that have high allele frequency differences.

## Results

### Differences in Methylation Between Life Histories

To test if epigenetics played a role in the adaptation of *A. alosa* to a complete freshwater life history, we sequenced 24 individuals of the Castelo de Bode freshwater population and 24 anadromous individuals caught in Rio Tejo (TEJ), the founder population of the Castelo de Bode (CBD) dam (Sabatino et al. 2021). Our in silico digestion suggests that the reduced genome corresponds to ~ 5.8% of the total genome size (~ 50 Mbp of 865 Mbp). On average, we sequenced at a depth of 32 × of the reduced genome (average ~ 30 × for CBD and ~ 34 × for TEJ). After adapter trimming and quality filtering, an average of 10 million reads were retained per sample. Samples with coverage lower than 10 × after quality filtering were removed from the analysis (6 from TEJ; 5 from CBD), resulting in a final set of 18 samples from TEJ (10 females; 8 males) and 19 from CBD (5 females; 14 males).

Our sliding window analysis yielded 20,680 windows of 2000 bp containing ten cytosines in CpG context in at least 10 individuals (more than half of the individuals in each population) with a minimum coverage of 3 per cytosine per individual. We performed a scaled PCA to evaluate these windows’ discriminatory potential. While no separation of the two populations was visible in PC1 (22.59% total variance explained), PC2 (9.87% of the variance explained) differentiated the anadromous and landlocked populations (Fig. 1B). Of the 20,680 windows analyzed for differences in methylation between anadromous and landlocked, 227 were significantly differentially methylated between life histories (q-value < 0.05; Δ-percentage-methylation > 25%). However, no region completely separated the two populations, with some overlap in the methylation percentage between individuals from both life histories (Fig. 1C). Of the 227 DMRs, 119 regions were hypermethylated and 108 hypomethylated in the CBD (Fig. 1C and D, Supplementary Table 2). These differentially methylated regions tended to be well distributed across the entire genome, with no apparent enrichment in any chromosome (Fig. 1D). On average, these windows were located 4,427 bp away from gene features and overlapped intergenic (40%), intronic (25%), promoter (19%), and exonic (16%) regions.

### Functional Enrichment Analysis

**Fig. 2:**
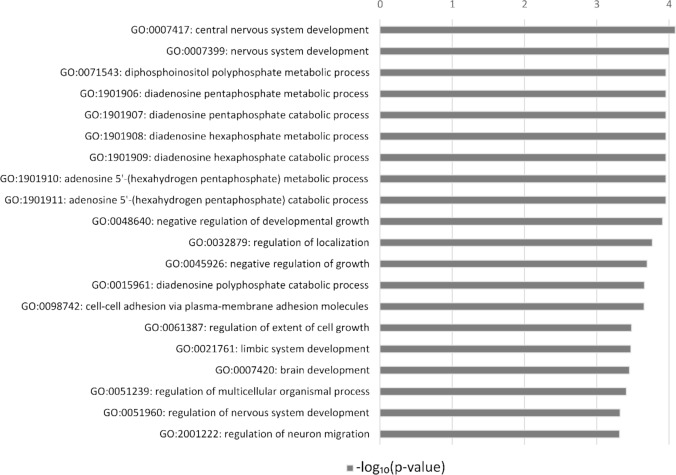
Top 20 GO categories for genes associated with DMRs. The X-axis shows the enrichment of GO terms using the Wallenius distribution –log10(p-value). Only categories with more than one differentially methylated gene were considered

To summarize the possible phenotypic consequences of epigenetic differences between landlocked and anadromous populations. To achieve this, we conducted a GO terms enrichment analysis for the genes found in differentially methylated regions. The top enriched categories (Supplementary Table 3) were correlated with brain and cranium development (GO:0007420; GO:0060322), central nervous system development (GO:0007417; GO:0007399), and metabolic and catabolic processes. These included the catabolic and metabolic processes of diadenosine, pentaphosphate, and hexophosphate (GO:1,901,906; GO:1,901,907; GO:1,901,908; GO:1,901,909), which have been implicated as inhibitory ligands of myocardial ATP-sensitive K + channels (Jovanović & Terzic 1995; Song et al. 2009). We also saw enrichment for molecular functions such as calcium ion binding (GO:0005509). However, while the above categories show a statistically significant *p-value*, the FDR-adjusted *p-value* exceeds our alpha threshold of 0.1, most likely due to the small number of differentially methylated genes. We, therefore, limit our report to the top 20 biological categories that show enrichment (Fig. 2). The complete list of enriched categories represented by two or more genes is in Supplementary Table 3.

### Genomic Regions Under Selection and Associations with Methylation

To test if DMRs are also under genetic selection for adaptation to freshwater in landlocked populations, we replicated part of the study presented in Sabatino et al. (2022). To do so, we used PCAdapt to perform genomic scans to identify genomic regions (windows of 20 SNPs) likely associated with adaptation. We identified several genomic outlier regions associated with adaptation to freshwater (top 1% outliers: 1726, FDR < 0.05: 14,185 windows), which matches those previously recovered in Sabatino et al. (2022), including a very strong signal on chromosome 7 that overlaps *Axin1* and *ATPase-α1.1b*. *ATPase-α1.1b* is known to be involved in ion exchange and homeostasis and, therefore, likely to be vital for the freshwater adaptation in this species (Sabatino et al. 2022; Velotta et al. 2017). Of the 227 differentially methylated regions, only five overlapped with the top 1% genomic outlier regions (3 uncharacterized genes, WWC2-like gene, and nt5c2b). When including all outlier windows recovered by PCAdapt, 31 of the 227 DMRs overlapped. These include regions with a variety of functions, including signaling (*ppp1r9bb)*, calcium ion-binding activity (*ncab*), and DNA damage sensor (*ATR*), among others.

### Impact of 5mC Deamination

**Fig. 3:**
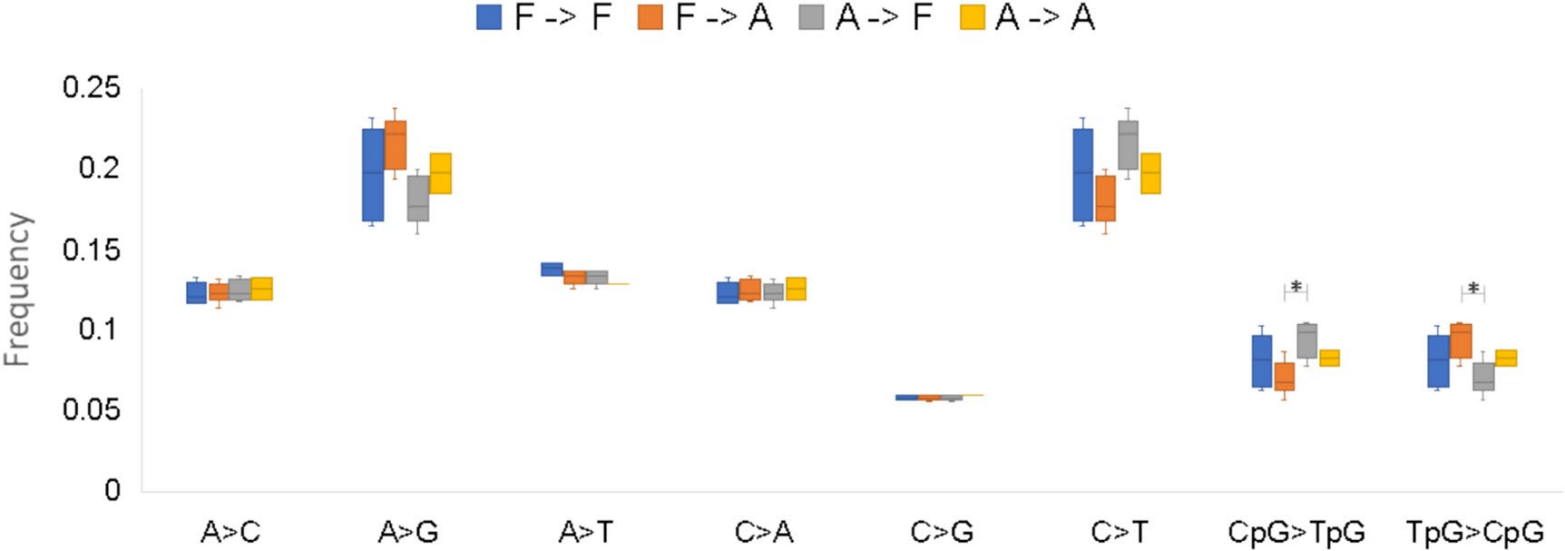
Directionality of the mutation spectrum for SNPs with a Δ allele frequency greater than 0.5. Interactions marked with a * are statistically significant (p < 0.05) using ANOVA followed by Tukey’s Honest Significant Difference post hoc test. F—> F shows the mutation spectrum between freshwater populations; F—> A from freshwater to anadromous; A—> F from anadromous to freshwater, and A—> A between anadromous populations

Methylation can impact the expression and function of genes in two ways. First, it can act as a regulatory signal, where the accumulation of 5-methylcytosines in the promoter or enhancer regions (but not exclusively limited to these) influences how the transcription factors bind to these, which can impede or promote expression (Yin et al. 2017). Secondly, methylation can impact gene expression through the deamination of 5-methylcytosines into thymine, which is the highest mutable motif in vertebrate genomes (Cooper & Krawczak 1989) and is associated with several diseases in humans (Cooper et al. 2010). To evaluate the influence of 5mC deamination to thymine in these populations. We found statistical support for differences between both transitions of CpG > TpG and TpG > CpG between freshwater and anadromous populations (with C > T and A > G transitions being slightly above our p-value threshold of 0.05). In these cases, an excess of deamination in 5-methylcytosines was observed from anadromous to freshwater (Fig. 3). This enrichment for TpG SNPs in the landlocked population is highly surprising as it suggests that the loss of CpG motifs in the freshwater population might carry a positive selective coefficient. However, given the recent age of the population, this positive selection is likely to act on standing genetic variation previously generated through mCpG deamination, rather than on de novo mutations.

We tested for evidence that the CpG-TpG transitions might have influenced our DMRs. We observe that only a small number of differentially methylated regions were linked with excess deamination (7 regions), suggesting that only a minority of the 227 DMRs detected were influenced by this mutation. However, due to how we validated windows for DMRs analysis, more precisely the requirement for a minimum of 10 CpG per window, some affected windows might have been discarded from the analysis, as CpG deamination might remove a potential site from an otherwise valid window. We, therefore, cannot completely exclude the effects of cytosine deamination in characterizing DMRs.

**Fig. 4:**
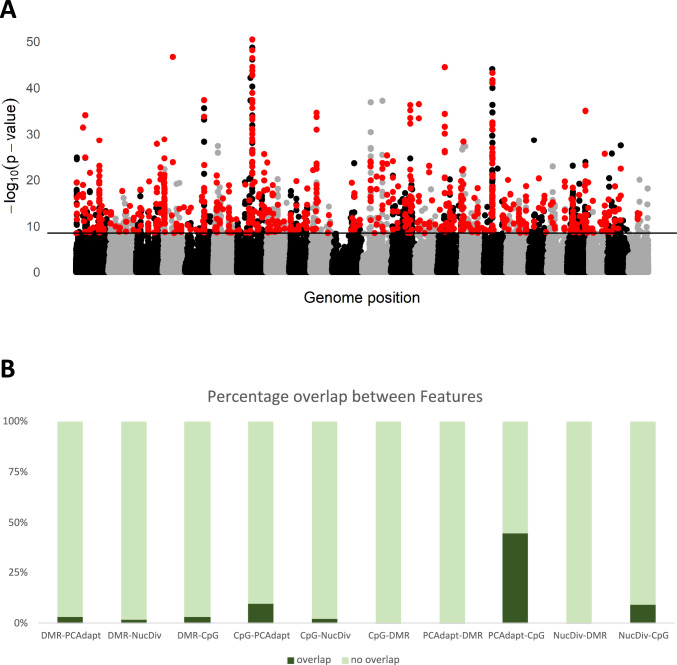
**A** Manhattan plot of –log10(p-values) of PCAdapt per genomic window of 20 SNPs. Black and gray points are for alternating chromosomes. Red points correspond to outlier windows that overlap with CpG- > TpG deamination. The horizontal black line corresponds to the top 1%. **B** Cumulative bar plots of the overlaps between significant regions across the multiple statistics. Due to differences in the number of outliers per statistic, we investigated how they behave in both directions. For example, for the interaction between PCAdapt outliers and CpG > TpG SNPs, the percentage of SNPs (13,970 SNPs) that overlap with the top 1% PCAdapt windows (1726 windows) is 9.4%. However, the percentage of PCAdapt outlier windows that overlap a CpG > TpG SNP is 62%. On the x labels: DMR – Differentially methylated regions; PCAdapt – Top 1% outlier windows from PCAdapt; NucDiv – Top 1% regions of the genome where Δπ is highest between life histories; CpG: CpG > TpG SNPs

Next, we tested whether putative regions under selection for adaptation to freshwater showed a strong correlation with CpG-TpG transitions. This could suggest a secondary role for DNA methylation, through mCpG deamination, in generating genetic diversity that could be recruited by selection. Notably, as expected, and in sharp contrast to what we see with DMRs, cytosine deamination is highly correlated with regions of the genome under selection. Of the 1727 top 1% windows, 771 overlap with one or more deaminated CpG (~ 45%; Fig. 4A and B). Furthermore, we evaluated if the degree of overlap between adaptive regions and cytosine deamination is just an indirect consequence of the higher nucleotide diversity in these regions by looking at the overlap of CpG-TpG transitions with regions of the genome with abnormal levels of Δπ. While there still is a high degree of overlap between the two, where 264 of the 1669 windows (top 1%) with the most extreme differences in π between life histories are associated with one or more deaminated CpG, the levels of overlap are comparatively lower (Fig. 4B). This difference between the enrichment of CpG-TpG transitions in outlier regions, compared to regions of high nucleotide diversity where the accumulation of mutations is expected, suggests that the enrichment in outlier regions is likely of biological relevance.

To test if relaxed control of DNA methylation due to the transition to freshwater is at the basis of the SNPs associated with the observed CpG- > TpG deamination events or if those SNPs are ancestral, we used π, θ, and Tajima’s D. Both π and θ are different measures of genetic diversity and. They can allow us to evaluate if a significant increase in nucleotide diversity is associated with the DMRs detected, which can be related to a relaxation of the selective pressure at these loci. Tajima’s D is a statistic that compares π and θ, allowing us to infer demographic/evolutionary processes affecting the different loci. In our analysis of π, we observed a slight increase in nucleotide diversity in both hypomethylated and hypermethylated relative to non-differentially methylated regions. However, differences between the two life histories were non-significant for all three categories, except when comparing hypermethylated regions with a minimum sample size and coverage of three. In all other, more stringent comparisons, there were no significant differences in nucleotide diversity between life histories for any of the categories; this scenario was closely mirrored by the results of our analysis for theta (see supplementary Fig. 2). As for Tajima’s D, our results show that both populations have mostly negative values. However, Tajima’s D tended to be more negative in the anadromous population (supplementary Fig. 2).

## Discussion

The landlocking of formerly anadromous *A. alosa* over the past century provides an excellent opportunity to study the molecular mechanisms involved in rapid adaptation. Previous work has demonstrated the importance of specific alleles or haplotypes of certain genes, such as ATPase-α1.1b, across landlocking events in Portugal (Sabatino et al. 2022). Here, we complement those results with evidence that genetic and epigenetic mechanisms were likely involved in the rapid adaptation to freshwater in these alosines. We identified differentially methylated regions neighboring genes with functions and physiological roles similar to those under genetic selection. However, we rarely encountered genes with both epigenetic and genetic changes. Furthermore, we found a strong interaction between CpG > TpG mutations and genomic regions under selection for freshwater adaptation. These results suggest that these previous deamination events might have created genetic variation that selection acted upon in this life history shift. Together, this study addresses some of the multiple ways by which populations can adapt to environmental change. Here, we discuss these results and their implications for understanding the tempo and mode of rapid adaptation in freshwater *A. alosa*.

### DNA Methylation in Adaptation to Freshwater

Our results suggest that DNA methylation plays an important role in regulating genes involved in adapting to freshwater in *A. alosa*. A recent review of recurrent patterns of adaptation to freshwater (Velotta et al. 2022) showed that selection tends to act on a set of genes, including active ion pumps, passive ion channels, claudins, and finally, hormones and their receptors. Several genes identified as under-selection in other species were here found differentially methylated between anadromous and freshwater *Alosa alosa* (Supplementary Table 2). Of those, we highlight several with functional roles that are consistent with metabolic changes known to be important in adaptation to freshwater: 1) ATP2B4, is one of the genes which encodes a plasma membrane Ca2 + ATPase that is responsible for the removal of bivalent calcium ions from cells against large concentration gradients. This particular subunit of ATP2B was also found under selection in the mummichog (*Fundulus heteroclitus* Linnaeus, 1766; Brennan et al. 2018), while other genes that encode different subunits of Ca2 + ATPase were found under selection in threespine stickleback (Hohenlohe et al. 2010; Jones et al. 2012a, b; Jones, Grabherr, et al., 2012b) and rainwater killifish (*Lucania parva* Baird & Girard, 1855; Kozak et al. 2014). 2) PRLH2 encodes for prolactin-releasing hormone and is related to the activity of prolactin and its binding with receptors. Prolactin is produced in the pituitary lactotrophs (Sakamoto & McCormick 2006), has multiple known roles ranging from immune response (Harris & Bird 2000) to reproduction (Whittington & Wilson 2013), and is important in the upregulation of the machinery that allows for ion transport in freshwater (Dobolyi et al. 2020; Manzon 2002; Sakamoto & McCormick 2006). Genes that code for this hormone or their receptors are under selection in the Atlantic herring (*Clupea harengus* Linnaeus, 1758; Martinez Barrio et al. 2016) and mummichog (Brennan et al. 2018). 3) KCNF1A and KCNH4B are Potassium Voltage-Gated Channel Modifiers (Subfamily H and F, respectively) with several functions. KCNF1A is part of the K_v_5.1 channels. These channels act as modulators of K_v_2 channels, which are responsible for maintaining membrane potential and electrical excitability in neurons and muscles (Gutman et al. 2005). KCNH4 is part of the K_v_12 channels that compose slowly activating voltage-gated potassium channels (Gutman et al. 2005), and its homolog (KCNH3) has been found to affect cardiac contractibility and cognitive performance in mice (Miyake et al. 2009). KCNH4 is also important for the adaptation to freshwater in multiple systems, including three-spined stickleback (Jones, Grabherr, et al., 2012b; Taugbøl et al. 2014), and its homolog KCNH1 has been associated with adaptation to freshwater in *Odontesthes humensis* de Buen, 1953 (Silveira et al. 2018). These examples suggest that DNA methylation might play an important role in the initial steps of adaptation to freshwater as a potential modulator of gene expression.

One important aspect of our results is that differences in DNA methylation between the two life histories did not always translate into a complete change in DNA methylation across all individuals within the two populations. For example, for most of the DMRs that show a high increase in DNA methylation in CBD, some individuals in TEJ also show an increase in DNA methylation. While at a different scale, a similar pattern was also observed in stickleback where for some differentially methylated cytosines, a few freshwater-adapted individuals showed a pattern closely related to that presented in the marine individuals (Hu et al. 2021). The adaptive response to freshwater likely involves multiple layers, including genomic architecture and the epigenome. These multiple layers can provide different avenues through which individuals can respond to the environmental change, however, with varying effects of fitness of each. Furthermore, the sampling of the anadromous population was already achieved in freshwater. Therefore, some of the DMRs here recovered may also be key for the migration to spawn carried out by the anadromous population. While the causes behind this overlap of individuals showing similar methylation responses in both populations can be difficult to dissect, it is likely the result of multiple factors, highlighting the complexity of adaptive responses.

### Functional Enrichment Analysis Shows Strong Enrichment of Growth and Brain Development Genes

Another key finding of our work is that differentially methylated genes between anadromous and freshwater shad are enriched for GO terms related to negative regulation of growth, head, and brain/central nervous system development (Fig. 2). While genes in these categories are unlikely to directly impact osmoregulation, they may reflect the morphological changes observed in these populations. It has been observed that even after a short number of generations, phenotypic differences begin to appear between the anadromous and landlocked life histories. For example, while anadromous males (6 years old) found in Portuguese rivers tend to measure between 58 and 64 cm, individuals of the same sex and age found in landlocked populations have become shorter, averaging 53.6–54 cm (Aprahamian et al. 2003). These changes might reflect a relaxation in selective pressures. The loss of the marine stage in the life cycle effectively eliminates the need for a growth period to accumulate the fat necessary to make the migration upstream during the reproductive season. Furthermore, it may also lead to a relaxation of some predatory pressures. Finally, freshwater populations no longer require the machinery that allows them to home during migration but must overwinter in difficult conditions (Correia et al. 2001), which might alter their brain function/development. Such changes in brain development have been observed in birds where migratory individuals tend to have a larger hippocampus, although overall smaller brain size when compared with residents (Mettke-Hofmann 2017). Our results indicate that, however, alosines have used different pathways, suggested by both genes associated with DMRs and their enriched GO categories, than those reported in three-spined stickleback for the adaptation to freshwater (Artemov et al. 2017; Heckwolf et al. 2020), and resemble more closely the enrichment for GO terms found between beach-spawning (Iteroparous) vs demersal (Semelparous) Capelin (Venney et al. 2022). However, no changes in parity are reported for the Castelo de Bode population, where individuals retain the ancestral semelparous trait (Aprahamian et al. 2003). Furthermore, the set of enriched GO categories is concordant with the genes previously found to be under selection in this system (Sabatino et al. 2022), and with categories found enriched in alewife (*Alosa pseudoharengus*, Wilson, 1811; Velotta et al. 2017). Overall, these results indicate that differences in methylation related to the adaptation to freshwater disproportionately involve genes associated with growth and brain development, which are likely essential traits for the species’ success in the new environment.

### Pitfalls of DNA Methylation

While our study suggests a role for epigenetics in freshwater adaptation in shad, as often is the case in epigenetic research, it is challenging to establish causality and independence from genetic information, especially in non-model systems. For example, we report little overlap between DMRs and regions with signals of natural selection (Fig. 4B), which could suggest a possible independence of these DMRs and genetic variation, excluding, of course, the effect of mQTLs that we do not have the power to detect. However, there are confounding factors that might influence the patterns described here. One source of such bias may be related to the sampling itself, as it is known that epigenetic patterns vary drastically between tissues and cell types (Zhang et al. 2013). While we made a strong effort to collect the same amount of tissue from the same area of the body across individuals, the cell populations collected and their proportions between samples will likely differ to some unknown extent. However, differences in cell types and their proportions can produce variability that could be detected as differentially methylated (Jaffe & Irizarry 2014; Lea et al. 2017). Indeed, we expect that differences in cell populations are driving the variance observed in PC1 (Fig. 1B). While this almost certainly increases variance and lowers our power to identify differentially methylated regions, it should be a random effect and should not impact our comparisons across life histories. Another source of bias concerns the choice of method to measure methylation. While RRBS is a well-established, cost-effective, high-resolution technique to measure genome-wide patterns of DNA methylation (Anderson et al. 2024; Artemov et al. 2017; Ghandi et al. 2019; Heckwolf et al. 2020), it also has several known drawbacks. For example, although the use of MspI to digest the genome ensures that most of the resulting sequencing library will contain CpG-rich regions of the genome that are typically associated with promoter regions (Boyle et al. 2012), it has the drawback that it significantly limits our capacity to evaluate differences in methylation in non CpG-rich regions. Furthermore, the reliance on a restriction enzyme can lead to some bias in the sites or genomic regions represented in the final dataset, thus impacting reproducibility (Carmona et al. 2017). Therefore, there might be other genes/regions involved in the adaptation to freshwater that we did not capture using our methodology.

One further potential confounding effect on our analysis is related to the samples’ age. While such a dataset allows us to obtain measurements of differences in DNA methylation just a few generations after the landlocking event, it is also true that sample’s age can have induced differences in DNA methylation that are unlinked to the underlying biological question. Indeed, a recent study aimed at identifying the stability of DNA methylation within cryo-preserved samples has demonstrated that while there are no observable global changes in the methylation profile, there is an apparent effect of sample age when comparing individual CpG sites, with a detectable loss of methylation (Lee et al. 2023). However, this effect tended to be small, affecting only a minute percentage (0.5%) of CpG sites (Lee et al. 2023). While we cannot assess how sample storage has affected the DNA methylation patterns here observed, as “modern” samples would also be affected by several other factors (e.g., population age; environmental conditions), we believe that, by using a large sample size, coupled with a likely stochastic effect of the loss of DNA methylation due to storage, the effects of sample age do not undermine the conclusions of our analysis.

### CpG > TpG Deamination is Highly Correlated with Regions Under Adaptive Selection

Our analysis has also shown that several regions of the genome that exhibited genetic selection signatures were also enriched for CpG deamination. Deamination of methylated cytosines in CpG context is the most frequent mutation in humans, with a mutation rate tenfold to 50-fold higher than any other transition (Cooper et al. 2010; Cooper & Krawczak 1989; Sved & Bird 1990). This C > T transition requires the methylation of the original cytosine. Otherwise, unmethylated cytosines produce uracil (U) when deamination occurs, which is removed by uracil glycosylase (Lindahl et al. 1997). This process carries several consequences for the maintenance of genes and regulatory function. First, the deamination of several CpG motifs creates regions of the genome with a higher bendability, while CpG-rich regions tend to be more rigid structures, especially when methylated (Basu et al. 2022). This change in DNA structure might promote differences in chromatin accessibility for gene expression and affect promoter–enhancer interactions (Lin et al. 2022). Second, CpG deamination creates transcription factor-binding sites with a much higher efficiency rate than other mutational events, promoting changes in the regulatory network of affected genes and potentially disrupting existing ones (Żemojtel et al. 2011). It can also directly impact enhancers where CpG deamination might create both gain and loss of function (Klein et al. 2018). Third, CpG deamination at promoter regions might change the methylation landscape and impact gene expression (Hernando-Herraez et al. 2015). Lastly, we report that not only does it seem there is an enrichment for SNPs resulting from CpG deamination in the transition from anadromous to a landlocked life history, but also that several genomic outlier regions in our genome scans are associated with these SNPs.

Indeed, it has been shown that there can be a correlation between genetic variations and DNA methylation patterns (Qu et al. 2012). However, due to the recent age of the population, it is a safe assumption that the genetic diversity resulting from CpG-TpG deamination is ancestral. This notion is supported by the fact that we do not observe an association between differentially methylated cytosines and increased nucleotide diversity. The CDB population we studied here was established just 50 years prior to our sampling. In contrast, the population of three-spined stickleback analyzed by Ord et al. (2023) is approximately 700 years old (Terekhanova et al. 2014), which might be sufficient time for relaxed selection to allow nucleotide diversity associated with DMRs to increase. These results suggest that genetic selection, in general, can be influenced by DNA methylation in several ways. For example, while mCpG sites tend to be depleted throughout the genome outside of CpG islands (Saxonov et al. 2006), it is possible that in the ancestral population of allis shad, some of these sites were maintained, either through balancing selection or neutral processes. These sites can then be selected and rise in frequency when populations undergo strong selective pressures, such as during transitions between life histories. This correlation indicates that the deamination of 5-methylcytosines, an indirect consequence of DNA methylation, might constitute a pathway to create standing genetic variation that can then be recruited during adaptation.

## Supplementary Information

Below is the link to the electronic supplementary material.

Supplementary file1 (PDF 232 KB)

Supplementary file2 (XLSX 121 KB)

## Data Availability

The raw RRBS sequence reads are deposited in the SRA (BioProject PRJNA1255642). Scripts used are available in a GitHub repository (https://github.com/PJADPereira/methylation_aalosa) and a snapshot will be kept through Zenodo (10.5281/zenodo.15540097).
