# Supplementary material for: Adaptation to Freshwater in Allis Shad Involved a Combination of Genomic and Epigenomic Changes
Source: J Mol Evol. 2025 Jun 2;93(3):406–22. doi: 10.1007/s00239-025-10253-9 (PMC12198289; doi:10.1007/s00239-025-10253-9)
Supplement: Supplementary file 1 — Supplementary file1 (PDF 232 KB) [file 239_2025_10253_MOESM1_ESM.pdf]

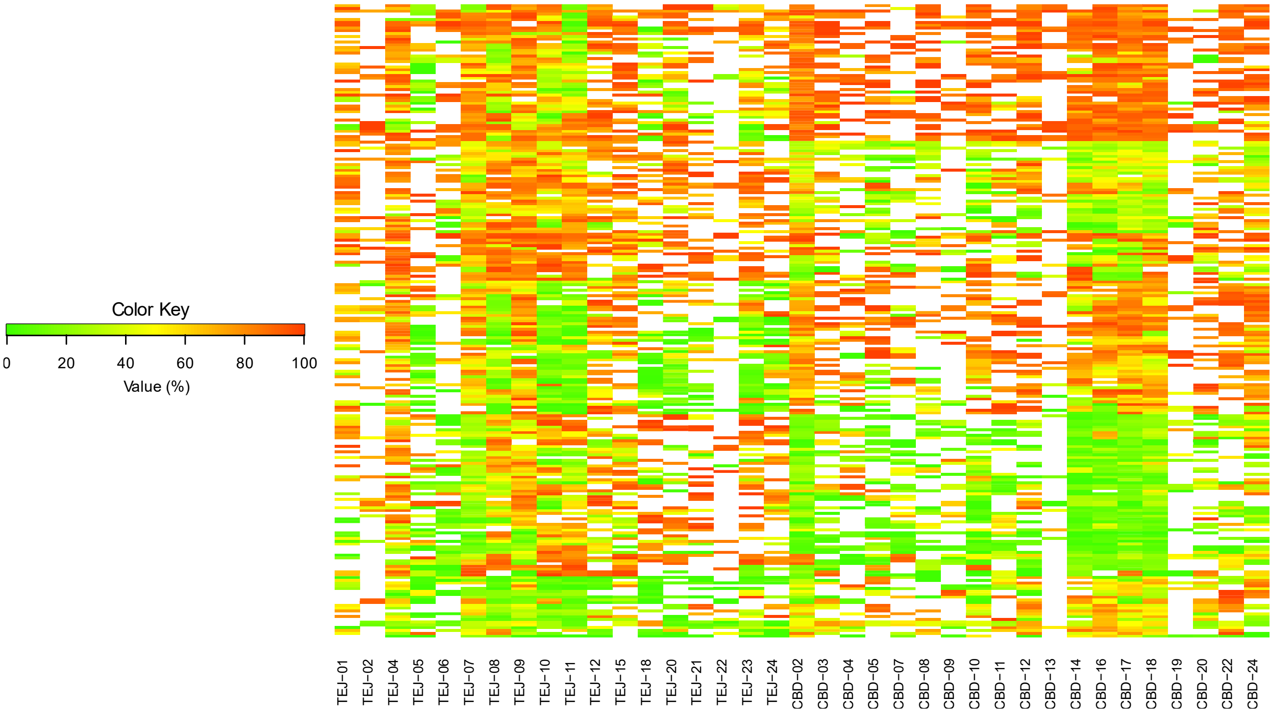

Supplemental Figure 1: Heatmap representation of the 227 differentially methylated regions (rows) identified in our study across all samples.

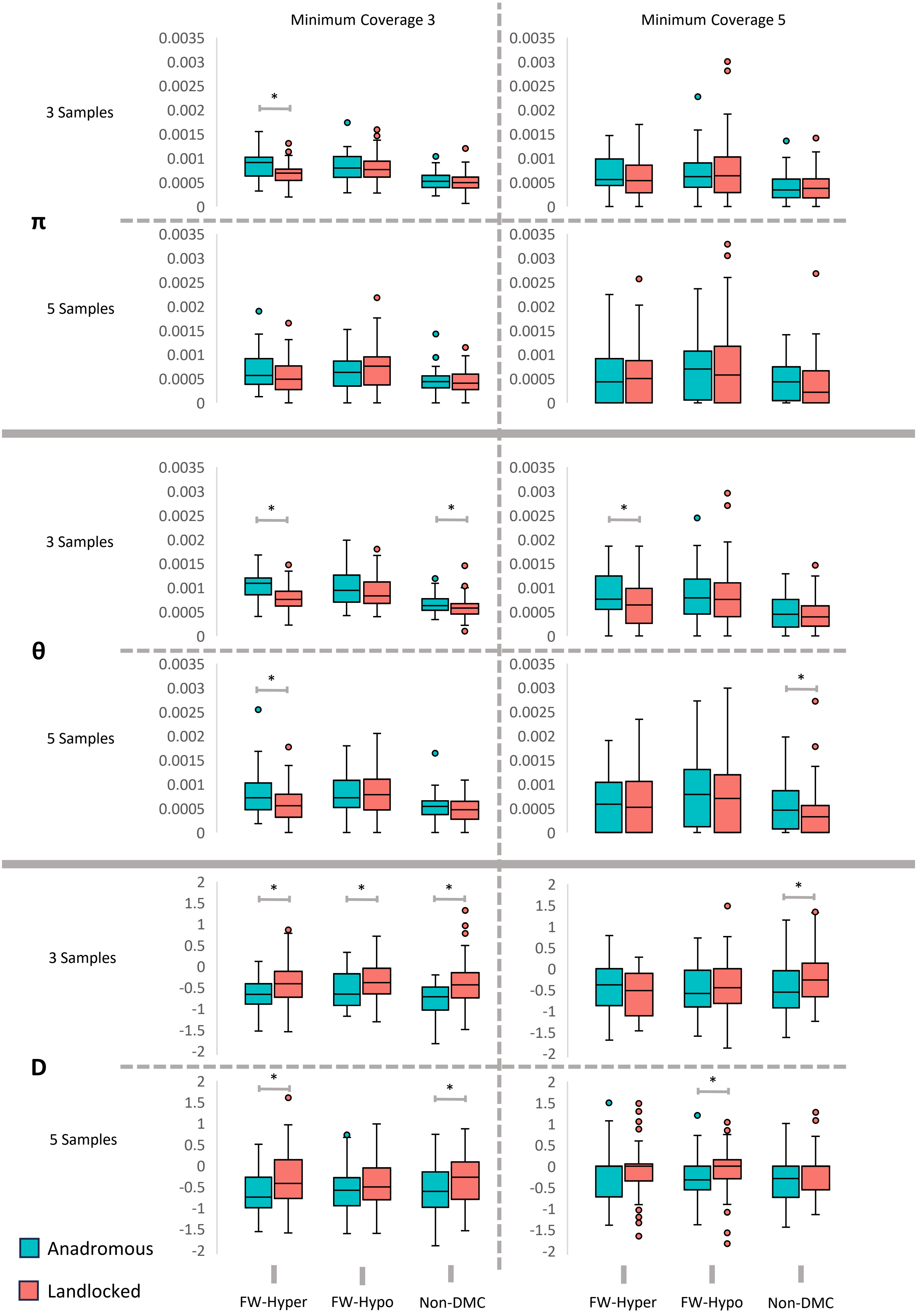

Supplemental Figure 2: Nucleotide diversity (π), theta (θ) and Tajima's D (D), for each life history, of methylated sites in different contexts, hypermethylated in freshwater (FW-Hyper), hypomethylated in freshwater (FW-Hypo) or not differentially methylated between the two populations (Non-DMC). The different thresholds tested and their impacts on nucleotide diversity are shown. Significant comparisons within a CpG context are marked with an *.
